# Design of CB-PDMS Flexible Sensing for Monitoring of Bridge Cracks

**DOI:** 10.3390/s22249817

**Published:** 2022-12-14

**Authors:** Yifeng Huang, Yugen Chen, Fangming Deng, Xiaoming Wang

**Affiliations:** 1 School of Civil Engineering and Architecture, East China Jiaotong University, Nanchang 330013, China; 2 School of Electrical and Automation Engineering, East China Jiaotong University, Nanchang 330013, China

**Keywords:** CB-PDMS composite, conductive ion glue film, compression strain, monitoring of bridge crack

## Abstract

This paper proposes a flexible sensor for detecting cracks on bridges. Strain and deflection sensing modules are integrated on the film that is made of composite conductive materials. By optimizing the preparation ratio and internal structure, the strain detection accuracy and sensitivity of the sensor have been improved. The bridge crack detection accuracy reached 91%, which is higher than current sensors. Experimental results show that the composite material containing 2.23 wt% carbon black (CB) mixed hybrid filler has good linearity, higher accuracy than sensors in use, excellent stretchability (>155%), high gauge factor (GF ~ 43.3), and excellent durability over 2000 stretching-releasing cycles under 10 N. The designed flexible sensor demonstrates the practicality and effectiveness of bridge crack detection and provides a feasible solution for accurate bridge health monitoring in the future.

## 1. Introduction

As a major concrete structure project and infrastructure, bridges are inevitably affected by loads and environmental erosion during use, and the damage they receive will cause structural resistance attenuation, sudden accidents, and huge casualties and property losses [1]. In order to ensure the safety, integrity, and durability of concrete structures, it is necessary to adopt effective technical means to detect cracks in concrete structures [2].

Concrete cracking sensors are important not only for bridges but also for concrete-made wind energy harvesting farms. In the past, crack detection mainly used a single fiber embedded point detection method in key parts of the structure [3]. However, due to the inhomogeneity of concrete materials and calculation errors, it is difficult to determine the angle between the embedded fiber and the crack [4]. The bonding strength and cracks of the concrete can cause permanent deformation of the optical fiber. Based on the digital image processing technology, the collected crack images are preprocessed to identify the edge information of the cracks so as to obtain the characteristics of the cracks. This method has high feasibility for the detection of cracks in a given local small area, but it is difficult to comprehensively detect small cracks in huge concrete structures and cannot effectively detect the location of cracks [5,6,7]. Piezoresistive materials are also widely used in the diagnosis and monitoring of cracks in concrete structures. However, a piezoresistive sensor that is embedded or pasted on the surface of the concrete structure cannot fully realize distributed detection, and the sensor will be damaged after the crack occurs, which will affect the subsequent detection performance [8].

Carbon black (CB) is composed of parallel-arranged graphitic planar bundles, an intermediate structure between graphite and non-three-dimensional structures, and the carbon atoms in carbon black particles are arranged in hexagonal planes forming a network of second-degree ordered planar layers. Most of the carbon black species are within the nanometer size range (<100 nm), and their surface is rich in reactive groups. The low-dimensional nCB is used as a conductive filler, meanwhile, PDMS with high elasticity and stability is used as a flexible matrix to prepare CB-PDMS flexible sensitive materials. The trapezoidal PDMS/nCB prepared by dissolution infiltration has high GF, high workable strain, high conductivity, low filler content, and excellent mechanical properties, reaching 355% workable strain at only 6.2 wt% CB loading [8]. With the rapid development of smart sensing materials, the use of flexible sensors that are made of sensing materials to detect concrete cracks has become a research hotspot [9]. Schumacher et al. used a carbon black (CB)-epoxy conductive composite material that was pasted on the surface of concrete to prepare a flexible sensor, and recorded the resistance change value through a fatigue load test, thus realizing effective monitoring of the formation and development of concrete strain, micro-cracks, and macro-cracks [10]. Based on a large-area electronic board that was composed of a dense array of unit strain sensors, Glisic et al. pasted the sensor array on a polyimide substrate and established a quantitative relationship between crack width and strain through a three-point bending test, thereby monitoring the occurrence of cracks and development [11]. Compared with traditional methods, flexible sensors have low cost, high accuracy, and high sensitivity for monitoring cracks, and it is easy to realize distributed monitoring [12,13,14].

The tension-sensitive flexible sensing material that was studied in this paper mainly uses its electrical properties (resistance and capacitance) to change with the change of tensile strain, and the relationship between the two can be used to monitor the deformation of concrete structures and changes, such as the occurrence and expansion of cracks [15]. When cracks appear in the concrete structure, especially when the cracks in the concrete structure develop to a certain extent (>0.4 mm), the detection of crack propagation becomes particularly important [16]. The flexible sensing material that was made in this research has good ductility and signal stability, it can achieve a high degree of conformal with the tested cracks, and it has been found through experiments that it has higher detection accuracy than other materials [17,18,19].

In this paper, using polydimethylsiloxane as the matrix and CB as the conductive carrier, CB-PDMS composite materials and conductive ion glues with low cost, high durability, high ductility, and good sensitivity were prepared, and the flexibility of the prepared materials was studied. The tensile sensitivity performance and mechanism of sensing materials, as well as the application of CB-PDMS composite materials to concrete deformation and crack perception characteristics, have theoretical significance and practical engineering application guidance significance for the preparation and application of new sensing elements in the field of concrete structure health monitoring.

## 2. Detection Principle and Structure Design

Cracks can characterize the stage changes of structural bearing performance and contain key information about structural hazards [20,21,22]. The timely and effective monitoring of the occurrence and expansion of structural cracks is of great significance for extending the service life of the structure [23,24,25]. In the actual bridge crack detection process, the traditional detection sensor cannot fit the crack closely, so there is a problem of low detection accuracy [5]. Aiming at the problems of existing detection methods, we have made a crack detection system that is based on flexible sensors that can meet the accuracy standards.

### 2.1. Sensor Module Design

The electrical properties and sensitivity of ionic polymers largely depend on the concentration of conductive fillers, the distance between filler particles, and the internal geometrical deformation under stress.

The characteristics of different carbon systems are shown in Table 1. CB has outstanding advantages such as good electrical conductivity, good tensile properties, and easy preparation, and compared with similar materials, the production cost is lower, so it is selected as the first choice for preparation. material [5]. At the same time, MBAA is added in the preparation to improve the conductivity of the conductive ion glue. Under similar load levels, the sheet resistance of the material that is doped with MBAA will be several orders of magnitude higher than that of the doped material. Then, the overall stiffness is reduced by reducing the Young’s modulus of the matrix and the conductive ion glue to enhance the elastic response of the flexible sensor under the condition of large deformation.

The resistance of a conductive composite is a functional equation between the resistance of the conductive particles and the resistance of the matrix. Since the conductivity of the conductive particles is much greater than that of the polymer matrix, the resistance through the conductive particles can be ignored [4]. When the conductive particles are far apart, no current flows. But when the distance between conductive particles is small enough, tunneling current will be generated. The following formula can be used to analyze the influence of tensile deformation and pressure on the resistance of conductive polymer composites. Zhang et al. [5] calculated the impedance of a single barrier. Considering the effects of all tunnel paths, the impedance of the composite material can be expressed by the following formula.

Simmons established a hypothetical model of filling silicone rubber with carbon black based on the tunnel effect theory, and proposed a universal Equation [6].

(1) $$J=\left[3{\left(2m\lambda \right)}^{1/2}/2d\right]{\left(e/h\right)}^{2}V\exp\left[-\left(4\pi d/h\right){\left(2m\lambda \right)}^{1/2}\right]$$

where *J* is current density, *h* is the Planck constant, *d* is gap width (nm), *λ* is barrier height (eV), and *m* is mass of 1 electron (g). It can be seen from the above equation that there is an obvious exponential function relationship between the tunnel current density and the gap width. Therefore, the quantum tunneling effect occurs only when the distance between adjacent conductive particles is small to a certain extent. The distance is too large and there is no electronic transition, that is, the conduction behavior of no current.

Considering that the application of uniaxial pressure will reduce the tendency of particles to separate and increase the possibility of a tunneling effect, the piezoresistance of the composite material with quantum tunneling effect is [7]:

(2) $$R=R_{0}\left(1-\frac{p}{G}\right)\exp\left(-4\pi \sqrt{\frac{2m\varphi }{h^{2}}}d_{0}\frac{p}{G}\right)$$

where *P* is applied pressure (MPa), *G* is compression modulus of composite material (MPa), *R_0_* is conductive particle resistance (Ω), *m* is mass of 1 electron (g), *h* is the Planck constant, *d_0_* is gap width (nm), and *φ* is percolation index.

On the other hand, the piezoresistive composite material that is based on the tunnel conduction mechanism also shows a huge resistance change when under tension. As the polymer matrix is usually hardly compressed or not compressed at all when stretched in one direction, the volume of the composite material remains constant in the direction perpendicular to the applied load. This effect causes the distance between the particles to decrease in the direction perpendicular to the applied stress [8]. As the conductive particles are randomly dispersed in the polymer matrix and are not perfectly aligned in the plane, deformation in the vertical direction of stretching will result in a redistribution of conductive particles in the composite, which will also create tunneling paths in the specimen [9]. The resistance of the test piece decreases exponentially with the applied stress, which can be expressed by the following formula [10]:

(3) $$R=\frac{R_{0}}{2\sqrt{1+\frac{p}{G}}}\exp\left(-2\gamma \frac{\sqrt{1+\frac{p}{G}}-1}{\sqrt{1+\frac{p}{G}}}\right)$$

where *γ* is percolation coefficient of composite materials.

Ezquerra and others deduced the relationship between the resistivity of the composite material and the distance between the tunnels of conductive particles [11]:

(4) $$\sigma =\sigma_{o}\exp\left[-{\left(2mV/h^{2}\right)}^{1/2}d\right]$$

where *d* is conductive particle tunnel gap (nm) and *V* is gap barrier (eV).

### 2.2. Manufacturing Process

**Figure 1:**
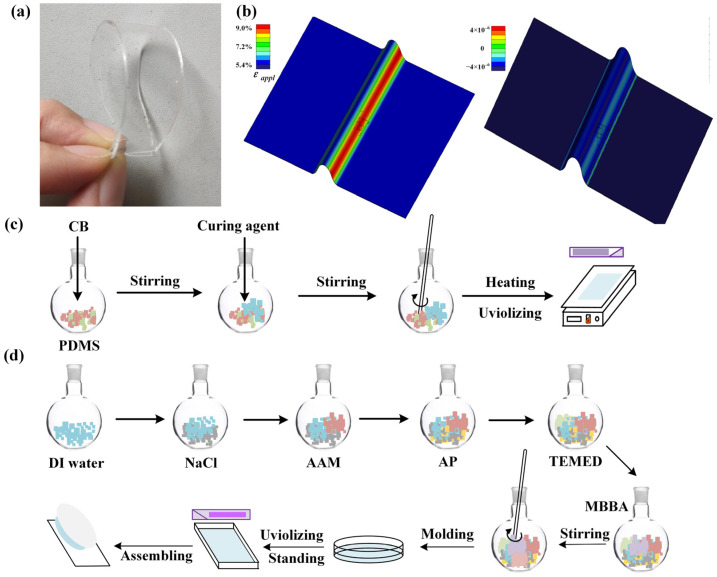
(**a**) The fabricated conductive film; (**b**) finite element analysis of structural crack perception characteristics (left: pressure intensity; right: displacement changes); (**c**) fabrication process of flexible substrate; and (**d**) fabrication process of conductive ionic glue.

We stirred CB into PDMS to produce the specimen to make a flexible sensor with good extension and stretch characteristics. The flexible sensing film that was made is shown in Figure 1a. It has a three-layer structure, namely the top layer, the middle layer, and the bottom layer. The top and bottom layers are substrates made of CB-PDMS, and the middle layer is a stress and pressure change sensing layer that is made of conductive ion glue. Perception ability is shown in Figure 1b.

1. CB-PDMS composite preparation

The specific preparation method is as follows. In order to prepare a substrate that meets the requirements, first the colloidal CB-PDMS (CB 2.23 wt%) was mixed and stirred for about 1 min, then the corresponding curing agent was added; the ratio of the two was 10:1. The mixture was stirred for 30 s and precipitated. The mixture was then flat coated on the metal plate. Finally, it was transferred to a heater and heated for 30 min with a temperature of 75 °C. The specific preparation process is shown in Figure 1c.

2. Conductive ion glue preparation

The preparation process of the conductive ion glue is shown in Figure 1d. The specific steps are. A total of 25 mL of deionized water had 3.91 g of acrylamide (AAM) (Aladdin Co., Ltd., Shanghai, China) added and 4.05 g of sodium chloride (NaCl) (Aladdin Co., Ltd.) and was stirred until the powder is completely dissolved. Then, 0.0425 g ammonium persulfate (AP) (Aladdin Co., Ltd.) and 0.0625 g N,N,N,N-tetramethyldiamine (TEMED) (Aladdin Co., Ltd.) were added to the mixed solution. The mixture was stirred, and finally 0.03 g of N,N-methylenebisacrylamide (MBAA) (Aladdin Co., Ltd.) was added as a cross-linking agent to maintain electrical activity, and set aside after fully stirring.

## 3. Calibration Process and Experiment

### 3.1. Calibration Experiment

The properties of flexible piezoresistive sensors are prone to be affected by factors such as temperature and material differences. To reduce the experimental error from individual differences, the calibration experiments were carried out in a simulated environment to ensure the authenticity and reliability of the output data.

Assuming that the mechanical properties of entire sensing array are the same, the tensile strain is shown as [12]:

(5) $$\gamma =\frac{l}{2d}$$

where *l* is the sample thickness and *d* is the bending radius. The different bending radius will change the resistance, which is:

(6) $$\frac{\left(R-R_{0}\right)}{R_{0}}=G\frac{l}{2d}$$

**Figure 2:**
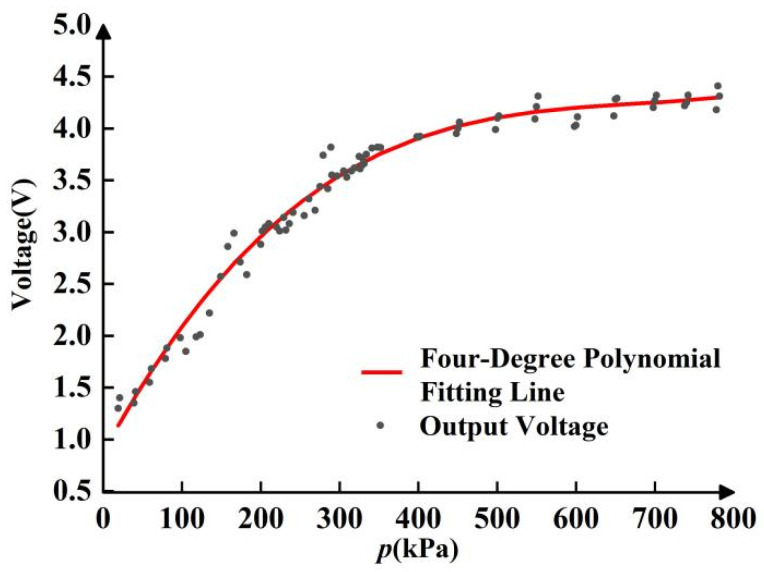
Fitting curve of the calibration experiment.

The calibration system consists of a loading platform, a data acquisition and transmission circuit, a power supply, and a computer visualization system. Electronic pressure gauges are used to apply pressure to materials and measure them through the designed visual graphics system to detect and derive the voltage. The operating pressure of the calibration experiment varied from 20 kPa to 800 kPa at room temperature (20 °C). After the data were collected and the image curve was drawn, the curve was fitted by a fourth-order polynomial equation, as shown in Figure 2.

### 3.2. Electrical Performance

**Figure 3:**
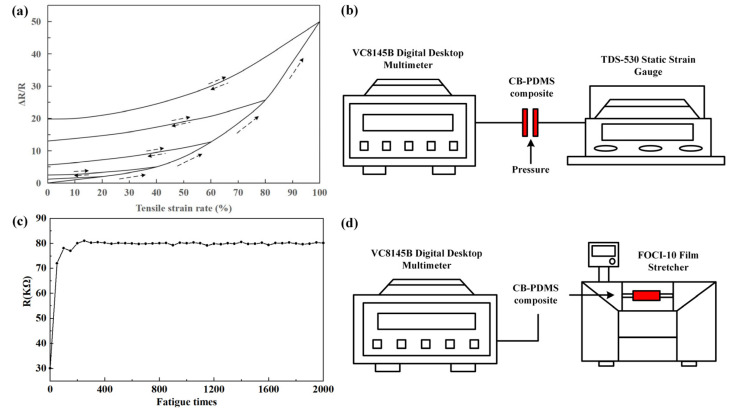
(**a**) Resistance changes of transparent conductive ion films in the range of 0~100% tensile strain; (**b**) experimental instrument of tensile strain monitoring; (**c**) resistance of conductive ion glue thin film via 2000 cycles of stretching and releasing at a certain tensile strain of 60%; and (**d**) experimental system of stretch properties monitoring.

The most important thing in the study of the performance of the transparent conductive ion glue film that was prepared in this section is its tensile sensitivity. This will determine to a large extent the transparent conductive ion glue film as a sensing material in monitoring concrete structure cracks, etc., as well as feasibility in practical engineering applications. In this section of the test, the resistance of the transparent conductive ion glue film changes with the tensile strain under uniaxial tensile strain, which is to test the tensile sensitivity of the transparent conductive ion glue film (Figure 3a). The entire test is performed under constant room temperature conditions. The experimental system is shown in Figure 3b.

Figure 3c depicts the resistance of the transparent conductive ion glue film under fatigue tensile strain (*ε* = 60%). After the conductive ion glue film undergoes 2000 fatigue stretching, the resistance of the transparent conductive ion glue film increases to 2.7 times the initial resistance. The main reason for the increase in the resistance is that as the stretching increases, the distance between adjacent carbon nanotubes increases, and the number of conductive connection channels between adjacent carbon nanotubes and the chance of electron transition is reduced, which directly leads to the connection resistance. Therefore, the resistance of the transparent conductive ion glue film also increases. The experimental system is shown in Figure 3d. 

### 3.3. Mechanical Behavior

**Figure 4:**
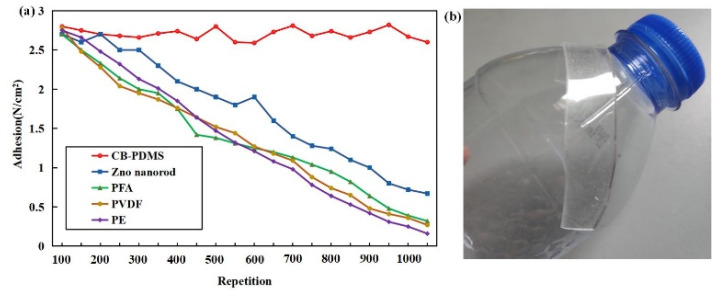
(**a**) Cyclic adhesion properties of the CB-PDMS (red), and other flexible pressure materials and (**b**) the sample glued to the plastic bottle.

In order to study the adhesion of CB-PDMS to bridge cracks, a tensile machine (MCT-2150, A&D Company, Tokyo, Japan) was introduced in the mechanical performance test. We set the tensile force to 10 N and the speed to 200 mm/min and then performed 1000 repeated adhesion-separation experiments on the cylindrical metal. The results are shown in Figure 4a.

We adhered the substrate to the PE material and found that it fits perfectly on the curved shape (Figure 4b). We made Zno nanorod powder (Aladdin Co., Ltd.) into films by hydrothermal methods, meanwhile, we have purchased PEA, PE, and PVDF films (LeanStar Co., Ltd., Suzhou, China) for control trials. After several adhere-detach experiments (Figure 4a), the adhesion of Zno nanorods decreased from 2.7 N/cm^2^ to 0.6 N/cm^2^, and the adhesion performance of other materials also decreased significantly, while the adhesion of CB-PDMS almost remained constant; it stayed around 2.7 N/cm^2^. The results show that CB-PDMS has a relatively better adhesive effect than the other tested materials. The GF of ~43.3 remained almost constant during the adhere-detach experiments.

According to the experimental results, when the thickness of the flexible sensor is 0.5 mm, the fracture rate is 81%, and when the thickness is 1 mm, the fracture rate is 115%. When the thickness is less than 0.5 mm, the tensile fracture rate is less than 20% and the tensile properties are not satisfactory. When detecting bridge cracks, the maximum stretch rate is about 55%, so it meets the tested stretch conditions.

### 3.4. Tensile Test

In order to verify whether the fabricated flexible sensor is suitable for a deforming environment, a tensile test was performed on the sensor. The performance of the CB-PDMS focuses on its tensile properties, the merits of which largely determine the feasibility for practical engineering applications such as monitoring cracks in concrete structures. Therefore, we carried out static loading experiments to test the tensile properties. 

**Figure 5:**
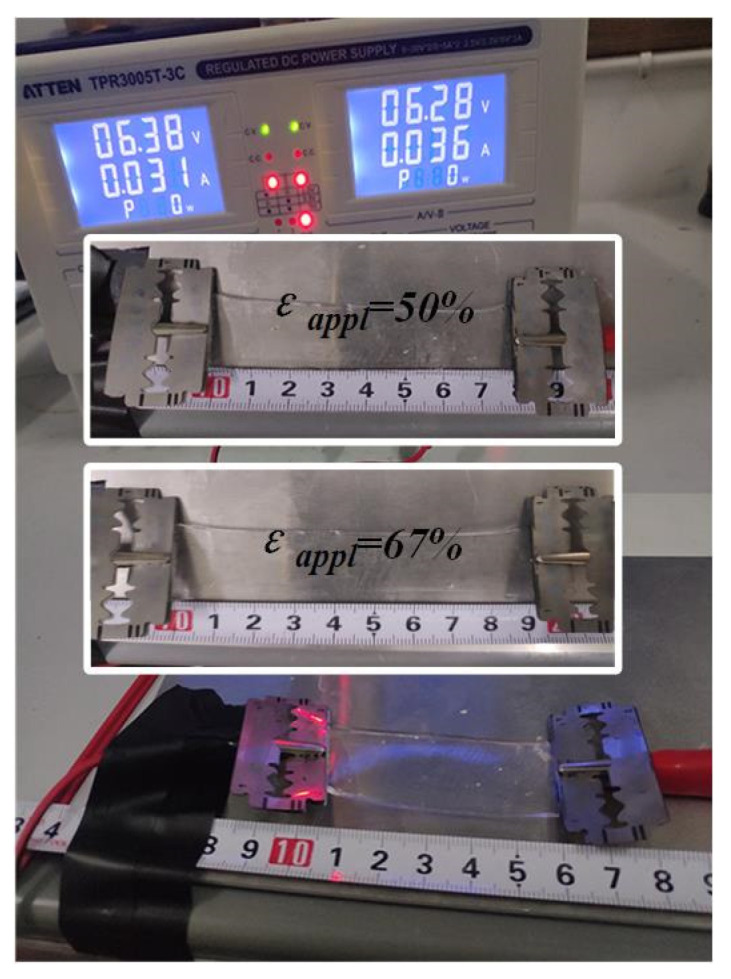
The sample was pulled off under different tensile forces, three sets of experimental scenarios at different stretch rates are shown, *ε*_applied_ represents the tensile rate.

A tensile machine (MCT-2150, A&D Company, Tokyo, Japan) was used to apply a tensile force to the fabricated sensor and record the data to obtain its elongation at break. The experiments were carried out at room temperature (25 °C) with a specimen size of 6 cm × 2 cm × 1 mm, shown in Figure 5.

The diagram shows that the specimen has good tensile properties, which remained unbroken when *ε_applied_* reached 67%. The experimentally measured tensile fracture rate was 82%.

### 3.5. Temperature Compensation

When the ambient temperature rises, the electron migration rate increases and the chance of transition increases, which is manifested macroscopically as an increase in the conductivity of the conductive composite material. Then, the increase in ambient temperature will directly cause the conductive composite material to be heated and deformed. If the conductive composite is heated to expand, the large difference in the thermal expansion coefficient between the polymer and carbon nanotube leads to the destruction of the internal conductive path of the material, which is manifested macroscopically as a decrease in the conductivity of the conductive composite material.

**Figure 6:**
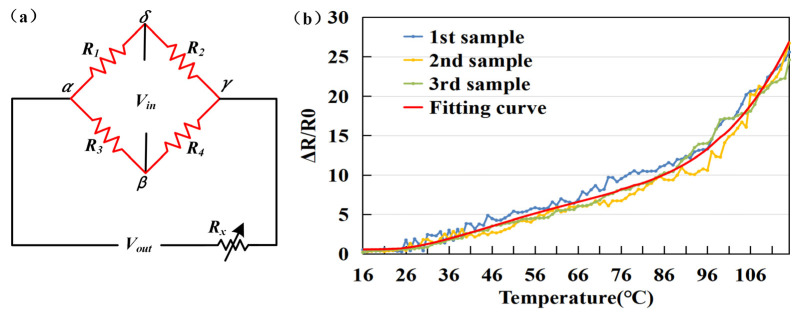
(**a**) Wheatstone bridge schematic diagram and (**b**) the relationship between temperature and resistance.

For the nonlinearity errors that occur and eliminating the influence of temperature on the measurement accuracy of the sensor array, we use the sensor bridge circuit feedback compensation method. The specific experimental methods are as follows. A Wheatstone bridge and compensation circuit were used for temperature compensation, then a thermistor and potentiometer were connected in the operational circuit to remove large temperature drift of sensitive components. The *V_out_* of a Wheatstone bridge (Figure 6a) is presented by Equation (7).

(7) $$V_{out}=\left(\frac{R_{1}}{R_{1}+R_{2}}-\frac{R_{4}}{R_{3}+R_{4}}\right)V_{in}$$

It can be seen from the experimental data that temperature and resistance are roughly positively correlated. After the curve is fitted by least squares, the fitted curve in the range of 15 °C to 110 °C has a good correlation (*R*^2^ = 0.98), the specific comparison is shown in the Figure 6b.

## 4. Structural Crack Sensing Detection Experiment

### 4.1. Compression Deformation Perception Characteristics

**Figure 7:**
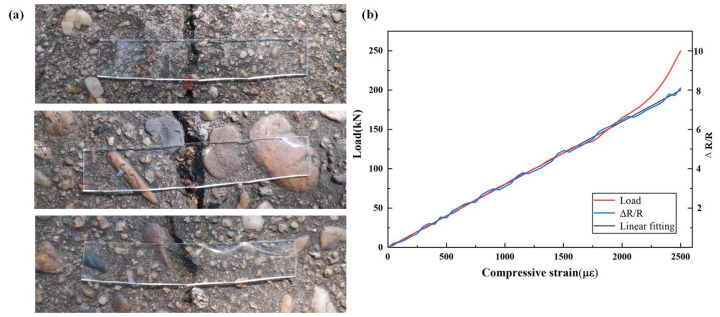
(**a**) Tests on samples with cracks and (**b**) the relationship between the resistance change of CB-PDMS composite and compressive strain of reactive powder concrete.

The monitoring of the concrete compressive strain under uniaxial compressive load conditions uses reactive powder concrete cube specimens measuring 60 mm × 80.5 mm × 40.5 mm. During the test, a 1000 kN universal pressure testing machine was used to apply the load, and the loading speed was controlled at a constant 0.2 MPa/s. The traditional strain gauge and CB-PDMS composite material specimens were horizontally pasted on the treated reactive powder concrete specimens with cyanoacrylate glue (Toclilee Co., Ltd., Dongguan, China). The position of the geometric center of a certain side is shown in Figure 7a. A ruler was used to mark the central area on the side of the test piece to prepare for attaching strain gauges and sensing materials. 

Among them, 0.02 mm thick copper sheets were used as electrodes to bond the two ends of the CB-PDMS composite with conductive silver paste. During the test, the electrode area was fixed with epoxy resin to eliminate the test errors that were caused by this part. During the test loading process, the pressure, strain, and resistance values were recorded synchronously, the compressive strain of the reactive powder concrete was collected by the FOCI-10 film stretcher [13], and the resistance value of the CB-PDMS composite material was collected in real-time by the VC8145B digital desktop multimeter, until the reactive powder concrete specimen was destroyed.

In order to better study the application feasibility of CB-PDMS composites in monitoring the compressive strain of reactive powder concrete under uniaxial compression, the relationship between the resistance change rate of CB-PDMS composites and the compressive strain of reactive powder concrete is used. The straight line fitting equation is obtained as Equation (8), and is shown in the purple straight line in the Figure 7b. In the fitting equation, the parameter α is −0.021, the parameter *β* is 0.21 × 10^3^, Δ*R*/*R* is resistance change ratio of CB-PDMS composite, and ε is compressive strain of reactive powder concrete [14].

(8) $$\Delta R/R_{0}=\alpha +\beta \varepsilon$$

Through the above test results and analysis, it can be obtained that the resistance change rate of the CB-PDMS composite material has a good quantitative relationship with the concrete compressive strain and has a good sensitivity coefficient. Therefore, similar to traditional strain gauges, CB-PDMS composites can be used as sensing materials to monitor the compressive strain of reactive powder concrete within the range of elastic deformation.

### 4.2. Bending Deformation Sensing Characteristics

To monitor the bending deformation of concrete, a reactive powder concrete specimen with a size of 40 mm × 40 mm × 160 mm was used, and the volume fraction of steel fiber was 1.5 vol%. The test adopts an MTS810 material testing system and a three-point bending loading method, where the distance between the two fulcrums at the bottom of the test piece is 100 mm, that is, the ratio of the span to the height of the test piece is 5:2, and the loading speed is controlled at 0.01 mm/s during the test. The CB-PDMS composite specimen was completely pasted on the central area of the treated concrete specimen area with cyanoacrylate glue (Toclilee Co., Ltd.). During the test, the pressure value, displacement, and resistance value were recorded synchronously, and the resistance value was recorded in real-time with a UT805A digital multimeter. The load was added until the concrete specimen is completely destroyed or the CB-PDMS composite material was broken.

In the three-point bending test of reactive powder concrete specimens, the upper surface of the specimen was subjected to pressure and the lower surface was subjected to tensile force. That is to say, as the stress continues to increase, the CB-PDMS composite material will also be subjected to tensile stress. As a result, the electrical resistance will also change, so there is a certain correspondence between the stress, deflection, and electrical resistance that is experienced by the reactive powder concrete specimens in the three-point bending test.

**Figure 8:**
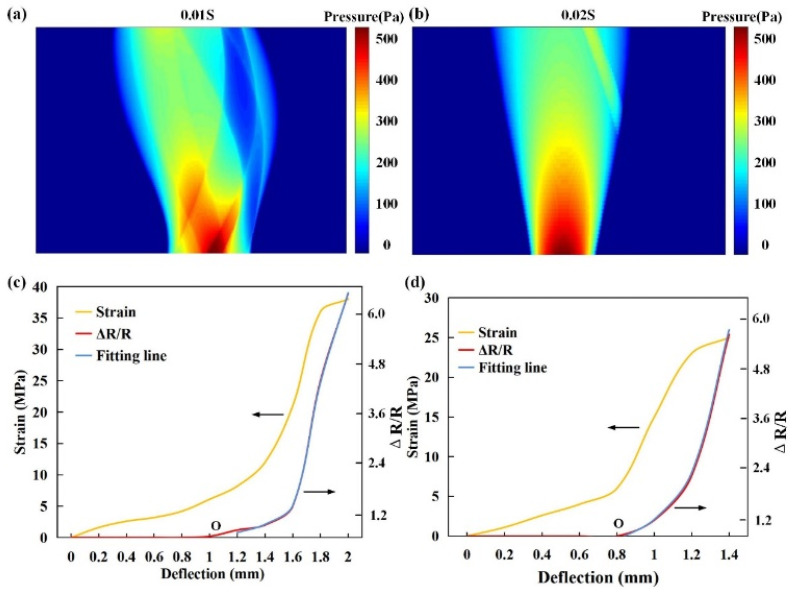
Distribution diagram of piezoresistive layer after being deformed by pressure in (**a**) 0.01 s and (**b**) 0.02 s. Relationship between resistance change of CB-PDMS composite and deflection of reactive powder concrete in (**c**) 10 wt% CB and (**d**) 15 wt% CB.

When the maximum stress value is reached, the reactive powder concrete specimen cracks, and the load continues to be applied. The pressure distribution diagram of a piezoresistive layer after being deformed is shown in Figure 8a,b with FEA. The red area is the part with a high strain and the blue area is the part with a low strain concentration. When the crack reaches a certain width, the CB-PDMS composite specimen fractures before the reactive powder concrete specimen. This is because the reactive powder concrete mixed with more steel fibers has a certain inhibitory effect on the development of cracks. Therefore, in the three-point bending test, when the resistance value of the CB-PDMS composite material became infinite, that is, after the composite material broke, the test was terminated and the crack observer was used to read the crack width value of the reactive powder concrete specimen at this time. The distance between the two pivot points at the bottom of the specimen is 100 mm, the ratio of span diameter to specimen height is 5:2, and the loading speed is controlled at 0.01 mm/s during the test. We recorded the strain, displacement and resistance simultaneously during the experiment. The load was applied until the concrete specimen was completely damaged or the composite material was fractured.

Analyzing the curve in the Figure 8c,d, it can be concluded that in the second stage (after O), the correspondence between the resistance change rate of the CB-PDMS composite and the three-point bending deformation of the reactive powder concrete is approximately exponential. The purple curve in the figure is the fitted curve. The specific fitting relation equation can be expressed by formula [15]:

(9) $$\Delta R/R_{0}=\alpha +\beta exp\left(\left(\delta -p\right)/\sigma \right)\;$$

where *δ* is deflection of reactive powder concrete specimen, the parameter *α* is −0.291, the parameter *β* is 0.241, the parameter *p* is 0.571, and the parameter *σ* is 0.127.

From the above test results, the resistance change rate of CB-PDMS composite material has a good corresponding relationship with the stress-strain curve of reactive powder concrete. Therefore, it is reasonable to monitor the bending deformation of reactive powder concrete and evaluate the damage degree of reactive powder concrete through the resistance change rate of composite materials. The performance of two literature sensors is compared with that in this paper, as shown in Table 2. The data proves that the fabricated sensor has good performance and meets the detection needs.

## 5. Conclusions

In the range of elastic deformation of reactive powder concrete, the relationship between the resistance change rate of CB-PDMS composite and the compressive strain of reactive powder concrete presents a good linear change in the initial crack generation and crack propagation stages of the reactive powder concrete, CB-PDMS composite. The relationship between the resistance change rate of the material and the deflection of the reactive powder concrete shows a good exponential change. CB-PDMS composites can be used as sensing materials to monitor the changes in compressive strain and deflection of reactive powder concrete in real-time.

In the three-point bending test, the initial change point “O” of the resistance value of the CB-PDMS composite can be used as the initial crack occurrence point of the reactive powder concrete. In the crack width range of 0.02~2.74 mm, the CB-PDMS composite can be used as a sensing material to monitor the occurrence and width expansion of cracks in polypropylene fiber concrete under the impact load of a free-falling ball. After using the CB-PDMS composite to determine the number of impact initial cracks, the two-parameter Weibull distribution can accurately and scientifically evaluate the impact life of PP fiber concrete.

## Figures and Tables

**Table 1:** Comparison of different carbon systems of conductive fillers.

Types	Classification	Conductivity (Ω^−1^·cm^−1^)	Tensile (MPa)	Disadvantages	Ref
Carbon System	CB	1.5 × 10^2^~6.2 × 10^5^	2.1~3.9	Likely to reunite	[1]
	CNTs	1.1 × 10^2^~1.8 × 10^4^	3.5~3.8	Complex for preparation	[2]
	Graphene	3.3 × 10^3^~1.9 × 10^3^	2.9~4.9	Instable conductivity	[3]
				Difficult to form chain aggregates	

**Table 2:** Comparison of current sensors for crack detection.

Types	Sensitivity	Response Time	Accuracy	Ref
Acoustic Emission Sensor	233.2 mV/V	500 ms	77%	[26]
Fibre Optic Sensor	−551 uV/V	1.2 s	82%	[27]
CB-PDMS Sensor	43.2 mV/V	2.1 s	91%	This paper

## References

[1] Perelmuter M. (2018). Analysis of interaction of bridged cracks and weak interfaces. Int. J. Mech. Sci..

[2] Valença J., Puente I., Júlio E., Arias-Sánchez P. (2017). Assessment of cracks on concrete bridges using image processing supported by laser scanning survey. Constr. Build. Mater..

[3] Dan D., Dan Q. (2021). Automatic recognition of surface cracks in bridges based on 2D-APES and mobile machine vision. Measurement.

[4] Peyton S.W., Sanders C.L., John E.E., Hale W.M. (2012). Bridge deck cracking: A field study on concrete placement, curing, and performance. Constr. Build. Mater..

[5] Adel M., Matsumoto K., Nagai K. (2021). Crack-bridging degradation and evolution in SFRC structural beams under variable amplitude flexural cyclic loading. Compos. Struct..

[6] Rypl R., Chudoba R., Scholzen A., Vořechovský M. (2013). Brittle matrix composites with heterogeneous reinforcement: Multi-scale model of a crack bridge with rigid matrix. Compos. Sci. Technol..

[7] Wang Q., Nakamura S., Okumatsu T., Nishikawa T. (2017). Comprehensive investigation on the cause of a critical crack found in a diagonal member of a steel truss bridge. Eng. Struct..

[8] Sun B., Xiao R.-C., Ruan W.-D., Wang P.-B. (2020). Corrosion-induced cracking fragility of RC bridge with improved concrete carbonation and steel reinforcement corrosion models. Eng. Struct..

[9] Su S., Yang Y., Wang W., Ma X. (2021). Crack propagation characterization and statistical evaluation of fatigue life for locally corroded bridge steel based on metal magnetic memory method. J. Magn. Magn. Mater..

[10] Tung P.-C., Hwang Y.-R., Wu M.-C. (2002). The development of a mobile manipulator imaging system for bridge crack inspection. Autom. Constr..

[11] Ye H., Duan Z., Tang S., Yang Y., Xu X. (2020). Fatigue crack growth and interaction of bridge wire with multiple surface cracks. Eng. Fail. Anal..

[12] Fu C. (2016). Dynamic behavior of a simply supported bridge with a switching crack subjected to seismic excitations and moving trains. Eng. Struct..

[13] Liu W.-H., Zhang L.-W., Liew K.M. (2020). Modeling of crack bridging and failure in heterogeneous composite materials: A damage-plastic multiphase model. J. Mech. Phys. Solids.

[14] Wu D., Wei M., Li R., Xiao T., Gong S., Xiao Z., Zhu Z., Li Z. (2019). A percolation network model to predict the electrical property of flexible CNT/PDMS composite films fabricated by spin coating technique. Compos. Part B Eng..

[15] Yu J., Chen Y., Leung C.K.Y. (2018). Micromechanical modeling of crack-bridging relations of hybrid-fiber Strain-Hardening Cementitious Composites considering interaction between different fibers. Constr. Build. Mater..

[16] Chlaihawi A.A., Narakathu B.B., Emamian S., Bazuin B.J., Atashbar M.Z. (2018). Development of printed and flexible dry ECG electrodes. Sens. Bio-Sens. Res..

[17] Afshar A., Daneshyar A., Mohammadi S. (2015). XFEM analysis of fiber bridging in mixed-mode crack propagation in composites. Compos. Struct..

[18] Cao J., Qin L., Liu J., Ren Q., Foo C.C., Wang H., Lee H.P., Zhu J. (2018). Untethered soft robot capable of stable locomotion using soft electrostatic actuators. Extrem. Mech. Lett..

[19] Hu N., Karube Y., Yan C., Masuda Z., Fukunaga H. (2008). Tunneling effect in a polymer/carbon nanotube nanocomposite strain sensor. Acta Mater..

[20] Li D.H. (2017). Three-dimensional analysis of transverse crack fiber bridging in laminated composite plates. Compos. Struct..

[21] Yin F., Ye D., Zhu C., Qiu L., Huang J.A. (2017). Stretchable, Highly Durable Ternary Nanocomposite Strain Sensor for Structural Health Monitoring of Flexible Aircraft. Sensors.

[22] Gong Y., Chen X., Tao J., Zhao L., Zhang J., Hu N. (2020). A simple procedure for determining the mode I bridging stress of composite DCB laminates without measuring the crack opening displacement. Compos. Struct..

[23] Nazmul I.M., Matsumoto T. (2012). Theory of inverse problems for crack bridging stresses determination. Int. J. Solids Struct..

[24] Selvadurai A.P.S. (2010). On the Mode I stress intensity factor for an external circular crack with fibre bridging. Compos. Struct..

[25] Kotoul M., Skalka P., Ševeček O., Bertolla L., Mertens J., Marcián P., Chawla N. (2017). Crack bridging modelling in Bioglass^®^ based scaffolds reinforced by poly-vinyl alcohol/microfibrillated cellulose composite coating. Mech. Mater..

[26] Curosu I., Liebscher M., Alsous G., Muja E., Li H., Drechsler A., Frenzel R., Synytska A., Mechtcherine V. (2020). Tailoring the crack-bridging behavior of strain-hardening cement-based composites (SHCC) by chemical surface modification of poly(vinyl alcohol) (PVA) fibers. Cem. Concr. Compos..

[27] Han Q., Li X., Xu K., Lu Y., Du X., Wang Z. (2021). Shear strength and cracking mechanism of precast bridge columns with grouted sleeve connections. Eng. Struct..

